# Derived datasets of daily weather, near surface soil status, flow rates and concentrations of nitrogen species from the North Wyke farm platform, England

**DOI:** 10.1016/j.dib.2025.111324

**Published:** 2025-01-22

**Authors:** Yusheng Zhang, Jane Hawkins, Hadewij Sint, Adrian L. Collins

**Affiliations:** Net Zero and Resilient Farming, Rothamsted Research, North Wyke, Okehampton, Devon EX20 2SB, UK

**Keywords:** Daily, Weather, Nitrogen, Water, Field scale

## Abstract

Weather conditions, hydrological responses and the dynamics of key nitrogen species in field runoff were continuously monitored at 15-min resolution on the intensively instrumented North Wyke Farm Platform (NWFP), a UK National Bioscience Research Infrastructure (NBRI), to support research on sustainable and resilient agriculture in the UK. Released data spanning 2013 to 2024 for 6 selected field catchments were aggregated to daily timestep, with reference to data quality flags, to produce continuous weather data, including maximum and minimum air temperature, daily total rainfall, wind speed and quality assured daily average soil moisture content, soil temperature at 15 cm depth, runoff rates, as well as nitrate, nitrite and ammonium concentrations. External data sources were sourced to infill some gaps for the weather data and summary statistics on data coverage were generated for the other data on an annual and seasonal basis where appropriate. Along with detailed field management data, the observed data provide a valuable resource for the parameterisation, calibration and validation of physically-based models for nitrogen losses at field scale to account for alternative management practices and land use under changing climate conditions.

Specifications Table

**Table:** 

Subject	Hydrology and water quality
Specific subject area	Daily timeseries of climate variables, soil moisture content, flow rates, nitrate, nitrite and ammonium concentrations.
Type of data	Table Filtered, Processed, Aggregated
Data collection	15-min data along with quality flags from a selection of field scale catchments were downloaded from the NWFP data portal (https://nwfp.rothamsted.ac.uk/) between July and September 2024. The targeted catchments comprise Pecketsford (catchment 1), Great Field (catchment 2), Poor Field (catchment 3), Burrows (catchment 4), Orchard Dean (catchment 5) and Higher Wyke Moor (catchment 8). These catchments were selected for their representativeness of land use types, similar hydrological contributing areas (∼6 ha), and relatively continuous data records. Data prior to 2013 were excluded to avoid the impacts of a catchment boundary change for catchment 4. Data quality flag-based filtering was undertaken to remove data records with potential issues. Data points with ‘Good’ and 'Acceptable' flags were accepted without change. Those with an 'Outlier' flag were checked individually. If multiple entries for the same timeslot were reported to have similar values, they were accepted. If an 'Outlier' value was reported for a single catchment then it was rejected. Data points with other quality flags were rejected out of caution. Daily totals, average values and sample counts from 09:00:00 were derived by creating pivot tables in Excel where adjusted date values were used as the row element. For weather data with <75 % of the expected data points (*n* = 96; i.e. 72), the data from a nearby Met office station (North Wyke site) were used. For wind speed, large gaps (>3 days) were infilled with the monthly average for valid wind speed readings. For temperature data with smaller gaps (<3 days), linear interpolations were used. For longer gaps (>3 days), gridded daily data from the HADUK-Grid dataset [1] were used. For rainfall data, gaps were handled with reference to the monitored flow data. If there was no detected flow, no rainfall was assumed. Otherwise, rainfall data from other sources were used; namely, observed data from the nearby Met station and daily data from HADUK-Grid.
Data source location	Institution: Rothamsted Research City/Town/Region: Devon County Country: England
Data accessibility	Repository name: Zenodo Data identification number: 10.5281/zenodo.14533996 Direct URL to data: 10.5281/zenodo.14533996
Related research article	None

## 1 Value of the Data

• There is a demand for quality observed data for the development and further refinement of process-based models for a better understanding of nitrogen cycling processes in agricultural landscapes to support evidence-based improvement of nitrogen use efficiency and mitigation of the unintended consequence associated with intensive farming [2]. Combined with detailed site descriptions and field management information which is publicly available, high resolution monitored data from the NWFP collected over recent years could provide unique reference datasets for representing agricultural activities in a UK setting.
• Using transparent and standardized approaches, the aggregation of 15-min data to daily time scale avoids the uncertainty associated with the pre-processing of data before their use in a process-based model. Access to data collectors, quality assessors and database administrators ensured appropriate interpretation of the quality flags. Local knowledge about alternative data sources ensured that the best available data were used for any necessary gap filling.
• Various data quality indicators, such as data coverage and valid data points for the daily estimates, will give potential model developers and users flexibility to select optimal years to use and to interpret any discrepancy between modelled outputs and observed values. These data series will be very useful for the testing of hydrological and water quality models in general with an emphasis on nitrogen losses from different land uses (low intensity livestock grazing with beef and sheep and arable cropping).
• Data collection spanned a period wherein arable cropping was introduced into a typical livestock grazing area in multiple field catchments and this coincided with recognized extreme wet weather conditions [3]. These unique combinations make the monitored data relevant to better process-based modelling of future climate change impacts under similar environmental settings.

## 2 Background

Process-based modelling has been an invaluable approach to improve our understanding of hydrological and nitrogen processes on agricultural land, which are key to sustaining various ecosystem services, including production of food and fibres, maintaining soil quality, and the filtering of harmful agri-chemicals delivered to aquatic environments. They are also the main tool for the assessment of potential impacts of alternative management practices under ever changing climate conditions. Most process-based models require large amounts of input data to run and they also need to be calibrated and validated before their intended applications. Existing models are mostly run at daily time step and require continuous weather data. There is a scarcity of monitored data at comparable temporal resolution for the testing of such models. Along with detailed field management information, the NWFP has accumulated a relatively long time series dataset at 15-min resolution for several field catchments [[5], [6], [7]] where some quality assurances have already been undertaken. Efforts were therefore made to further process the available data to make them suitable for the development and testing of physically-based nitrogen models for UK agricultural settings.

## 3 Data Description

The generated new data [4] are provided in an Excel workbook named ‘Derived daily outputs from 15 min NWFP data.xlsx’ which contains 6 separate worksheets with self-explanatory sheet names: ‘Weather’; ‘Soil moisture’; ‘Soil temperature’; ‘Flow rate’; ‘Nitrate concentration’, and; ‘Ammonium concentration’, respectively. The first cell of each sheet gives a brief description of its content. Two blocks of data are presented on each sheet: daily time series for 6 catchments on the left and summary statistics data on data coverage on the right. For the ‘Weather’ sheet, a column with a header of ‘Infilled variable list’ lists individual data items that are not from NWFP monitoring for each data record. The majority of the data records were based entirely on NWFP monitoring and they were registered as ‘Unchanged’ in the appropriate column. For the other datasets, the number of accepted data points available following filtering and used for the calculation of daily values are tabulated. For summary data on the temporal coverage throughout the reported period, annual percentages are given for all data series. In addition, percentage coverages for the soil drainage period (October to March) are also shown for nitrate and ammonium concentrations. A sheet named ‘Metadata’ is provided to give a brief description of the data processing procedures involved. Information for relevant field management events, including ploughing, drilling and harvesting, fertilizer application timings and rates, as well as manure spreading timing and rates, are shown on a field basis in a separate workbook named ‘Field management information for modelling.xlsx’. The membership of fields to the selected NWFP catchments is explained in a separate worksheet, named ‘Catchments and Fields’. A separate KMZ file (‘catchment boundary.kmz’) is included as part of the data package to show the geographic extent of the field catchments concerned, which is a widely used data format for spatial mapping. It can be opened in google maps, online free viewer (e.g., https://kmzviewer.com), or specialised GIS software (e.g., ArcGIS).

## 4 Experimental Design, Materials and Methods

### 4.1 Catchments and land uses

The monitored 15-min data are from 6 hydrologically-isolated catchments which form part of a purposely instrumented farm-scale study site or platform for the comparison of 3 farming systems referred to as farmlets (i.e. mini-farms): permanent pasture as a control (Green farmlet), increased use of legumes and replacement of chemical fertiliser (Blue farmlet) and planned reseeding and regular renewal/arable (Red farmlet) [5]. Catchments 1, 2 and 3 are part of the NWFP Red farmlet; Catchments 4 and 5 are part of the NWFP Green farmlet, and Catchment 8 is part of the NWFP Blue farmlet. Between 2013 and 2019, they were all used for low intensity sheep and cattle grazing. Catchments 1,2 and 3 were converted from pasture to the production of cereal crops in the autumn of 2019 wherein different crops (winter wheat, winter oats) have since been cultivated under different land management practices (conventional ploughing, minimum tillage). Further details can be found in the assembled field management information provided.

### 4.2 Instrumentation & sensors

Air temperature, wind speed, solar radiation and precipitation, were recorded at 15-min intervals using dedicated meteorological equipment (Adcon, OTT HydroMet GmbH, Vienna, Austria) sited at an approximately central location on the farm platform. The instruments were co-located next to those of an official UK Meteorological Office site which has collected daily data (9am – 9am), since 1981.

Air temperature was measured using a thermistor (Adcon TR1 Combisensor Air Temperature) with a range of −40 – 60 °C, wind speed was measured at a height of 3 m using an anemometer with a range of 1.44–270 km h^−1^, and solar radiation was measured using a pyranometer (range 0–1600 W m^−2^; resolution 0.1 W m^−2^). From 2013 - 2015, precipitation was measured by a tipping bucket rain gauge (Adcon RG1 Rain Gauge 200; range = 0 – 100 mm h^−1^; resolution = 0.2 mm) but this was replaced on 2015–03–16 with a more accurate Pluvio rain gauge (Pluvio^2^ L, Adcon, OTT HydroMet GmbH, Vienna, Austria) with a range of 0.1–500 mm h^−1^ and resolution of 0.01 mm. Although capable of monitoring precipitation data at 1-minute intervals, only 15-min interval data were recorded. Given that the instruments were co-located with those of the UK Meteorological Office, periodic comparisons were conducted to check for consistency between the alternative measurements and differences of genuine concern were flagged during the quality control (QC) processing of the data described below.

Soil moisture, at depths of 10 cm and soil temperature at 15 cm, were monitored at 15-min intervals from an approximately central location in each of the field scale catchments using a combined soil moisture (using capacitance) and temperature probe (A51760; A51730, Adcon, OTT HydroMet GmbH, Vienna, Austria). The probe is connected via an SDI 12 interface to a remote terminal unit (RTU, A723 addIT Series 4, Adcon, OTT HydroMet GmbH, Vienna, Austria) for data transmission. The scaled frequency unit (SFU) data were converted to % soil moisture (%) using the formula shown below:

soilmoisture=(SFU−18.80)/1.808

which is developed from calibrating the sensor output in 1m^3^ blocks of North Wyke soil under a range of different soil moisture conditions. During 2015, the A51760 model Adcon sensors were replaced with the A51730 model (catchment 1: 2015-09-16; catchment 2: 2015-08-24; catchment 3: 2015-08-24; catchment 4: 2015-02-04; catchment 5: 2015-09-16; catchment 8: 2015-07-11), and the data converted using an updated calibration formula:

soilmoisture=(SFU+12.87)/1.808

Visual examination of temporal patterns in the data at a daily time scale indicated that no step changes were introduced as a result of instrument changes.

Hydrological flow from each catchment was collected by two French drains on the downslope boundaries of the catchments that merged in a confluence pit. From here, flow was channeled via a conduit to H Type flumes (TRACOM Inc., Georgia, USA), the size of which was determined by size of the catchment they are servicing. The level or stage height of water was recorded at 15-min intervals using sensors sited in a stilling well near the flume outflow. Up until mid-2015, flow was monitored using bubble flow meters (4230, Teledyne ISCO, New England, USA). These were replaced with pressure level sensors (PLS500 Pressure Probe, OTT Hydromet, Loveland, CO., USA).

The level height (H) data are converted to flow (L s^−1^) using formulas specific to the size of the flume, and which are given in Table 1.

**Table 1:** Formulae for conversion of water height to discharge rate for different sized flumes.

Catchment Number	Flume size (ft)	Formulae (H in metres)†
1	1.5	L^-s^ = −0.00396436 – (0.07231968 * H^0.5^) + (79.89379128 * H^1.5^) + (900.3765227 * H^2.5^)
2, 3, 5, 8	2.0	L^-s^ = 0.022285358 – (0.55496382 * H^0.5^) + (125.5275778 * H^1.5^) + (939.5717311 * H^2.5^)
4	2.5	L^-s^ = 0.042446953 – (0.90725263 * H^0.4^) + (108.676075 * H^1.4^) + (937.5943603 * H^2.5^)

† Taken from Field Manual for Research in Agricultural Hydrology, Agriculture Handbook No 224, U.S. Department of Agriculture, February 1972.

A cabin sited at each flume contains telemetry devices for transmission of data via fibre optic cable, pumping equipment, and a custom-built stainless-steel by-pass flow cell (13 L capacity) that houses sensors to measure various water quality parameters at 15-min intervals. Water from a sump in the conduit that supplies the flume was automatically pumped into and out of the underside of the flow cell by a bi-directional peristaltic pump (621VI\R, Watson-Marlow Inc., Massachusetts, USA). The V-shaped design of the flow cell ensured that there was no build-up of sediment or particulate matter either between samples or over time. The pumping cycle was controlled through a combination of the level sensor data, a netDL 1000 data logger (OTT Hydromet, Loveland, CO., USA), and a programmable logic controller (PLC LOGO, Siemens AG, Munich, Germany). The programmable logic controller (PLC) stored a programme that activated the peristaltic pump, as well as controlling its speed and direction. The sensor level data were captured by the netDL logger, and a signal sent to the PLC depending on the flow conditions. If a 15-min flow point was ≥ 0.2 L s^−1^, the PLC programme was activated and the pump operated, but if the flow point was ≤ 0.18 L s^−1^, the PLC programme was de-activated, no pumping took place, and the volume of water in the flow cell was retained. This prevented sensors, such as ion elective electrodes (ISEs) that require permanent submersion in a liquid, from drying out.

Ammoniacal nitrogen (NH_4_^+^, NH_3_) was measured by an ISE (range = 0–100 mg L^−1^; resolution = 0.01 mg L^−1^) as part of a suite of sensors attached to a multi-parameter sonde. Up until May 2016, YSI 6600V2 sondes (Xylem Inc Rye Brook, New York, U.S), that also held sensors measuring other water quality parameters including turbidity, dissolved oxygen, specific conductivity, pH and temperature, were used. During 2016, the YSI 6600V2 sondes were upgraded to Xylem YSI EXO 2 sondes (Xylem Analytics UK, Letchworth, UK) fitted with smart sensors. The sondes communicated directly with the netDL logger via a Serial Data Interface.

Combined nitrate-N and nitrite-N were measured by a dedicated, self-cleaning, optical UV absorption sensor (NITRATAX Plus SC, Hach Company, Loveland, Colorado, USA) with a range of 0.1–100 mg L^−1^ and resolution of 0.1 mg L^−1^.

### 4.3 Water quality sensor calibration

Initially, two complete sets of the YSI 6600V2 sondes were used allowing one set to be calibrated and stored in the laboratory while the other set was deployed in the field. After the upgrade to the YSI EXO 2 sondes in 2016, the design allowed for smart sensors to be plugged in and removed easily. Therefore, two complete sets of sensors were used; one set was deployed in the field whilst the other was calibrated and stored in the laboratory, ready for deployment.

The sonde/sensor sets were rotated approximately every month, minimising downtime, and ensuring continuous high data quality. All sensors were checked in standards of known concentration and drift values recorded. Once drift checked, the sonde sensors were cleaned and stored appropriately until they were calibrated prior to deployment. In the case of NH_4_^+^/NH_3_ ISEs, a 2-point calibration (1 mg L^−1^ - 100 mg L^-1^) was used, and sensor modules were replaced every 12 months. Following storage, ISEs were re-hydrated by soaking for 24 h in a 100 mg L^−1^ NH_4_^+^ standard prior to calibration and deployment.

The Nitratax UV absorption sensors remained in situ and were calibrated monthly in the field using a 2-point calibration (0 mg NO_2+3_ L^−1^ (Reverse Osmosis water) - 11.3 mg NO_2+3_ L^−1^). Sensor drift that might be due to lens contamination was checked prior to cleaning the lens and wiper blade. In addition, the sensors underwent an annual service including a 3-point factory calibration.

For more detailed information on the design and set-up of the North Wyke Farm Platform (NWFP) and the instrumentation described above, please refer to relevant guide documents [[5], [6], [7]].

### 4.4 Quality control of data

A detailed description of the QC processing of the data can be found in Hawkins et al. [8]. Briefly, the QC process used custom-developed R (http://www.r-project.org) scripts on four weeks’ worth of data at a time. Each data point was given an appropriate flag to give an indication of reliability.

A Sensor Downtime Log (SDL) of all sensor issues was maintained including details on the sensor, its location, the start and end times the sensor was functioning incorrectly, information about the issue and the required QC action (i.e., set recorded data as missing (NA) or add an ‘unreliable’ flag to each data point). Exports from the SDL were automatically used as part of the QC process and based on the records, data were flagged unreliable for certain periods which could be from a few hours up to months. The QC process applied limits to identify extreme distributional (lower limit and upper limit) outliers, whilst other limits were used to identify simple distributional (lower limit and upper limit) outliers. The limits were not set statistically but were based on expert judgement of the data to identify unusual or interesting low- and high-valued measurements. Values exceeding extreme upper or lower limits, or deemed impossible, were set to NA. Thus, the assignment of flags was rather subjective and based on various events that have taken place (recorded in the downtime log), that might potentially have affected the data, without knowing the full extent of the issue or event. Data quality flags that might be assigned to a data point and their explanations are given in Table 2.

**Table 2:** Data quality flags – description and details.

Flag	Details
Not set	No information on quality available
Good	Data were checked and deemed good
Acceptable	Data were checked and no issues were found
Suspicious	Data were checked and might have been affected by an event
Highly Suspicious	Data were checked and have definitely been affected by an event
Reject	Data were rejected
High Sensor Drift	Calibration values indicate that the readings were high over the time period. As calibration takes place monthly, it is impossible to know if or how much the instrument drifted at the measurement timestamp as this is not a linear relationship
Missing Sensor Drift	Missing instrument calibration information, this level of instrument drift during the period is unknown
Outlier	The value falls outside ‘regular’ limits but within the extreme limits, therefore could still be fine
Level Reset	Level pressure sensors were reset, indicating this could result in a step change in flow
Calibration	Calibration Datetime of the instrument

## Limitations

Due to the impacts of Covid shutdown, the data coverage for some years is low. Generally speaking, there is also low coverage for crop growth periods (May to August) because of low/discontinuous flow conditions at field scale and the threshold flow-based water sampling regime. No comparable data on nitrogen related emissions to air, such as nitrous oxide, were available at similar temporal resolution or coverage. The data series assembled here do not contain all the related information on field and catchment conditions and land management practices; e.g., livestock management. Comprehensive management information is available from the open access NWFP data portal (https://nwfp.rothamsted.ac.uk/).

## Ethics Statement

Hereby, we (Yusheng Zhang, Jane Hawkins, Hadewij Sint, Adrian L. Collins) have declared that we have read and follow the ethical requirements for publication in Data in Brief and confirm that the current work does not involve human subjects, animal experiments, or any data collected from social media platforms.

## Credit Author Statement

**Yusheng Zhang:** Conceptualization, Formal analysis, Investigation, Methodology, Validation, Visualization, Writing –original draft, Writing –review & editing; **Jane Hawkins:** Investigation. Validation, Writing –original draft; **Hadewij Sint:** Investigation, Validation; **Adrian L. Collins:** Conceptualization, Validation, Funding acquisition, Project administration, Resources, Supervision, Writing –original draft, Writing –review & editing.

## Data Availability

ZenodoDerived daily timeseries of weather, soil moisture and temperature, flow and nitrogen species (nitrate and nitrite, ammonium) concentrations data for the North Wyke Farm Platform National Biosciences (Original data).
